# Multifunctional role of natural products for the treatment of Parkinson’s disease: At a glance

**DOI:** 10.3389/fphar.2022.976385

**Published:** 2022-10-06

**Authors:** Md. Mominur Rahman, Xiaoyan Wang, Md. Rezaul Islam, Shopnil Akash, Fatema Akter Supti, Mohona Islam Mitu, Md. Harun-Or-Rashid, Most. Nazmin Aktar, Most. Sumaiya Khatun Kali, Farhana Israt Jahan, Rajeev K. Singla, Bairong Shen, Abdur Rauf, Rohit Sharma

**Affiliations:** ^1^ Department of Pharmacy, Faculty of Allied Health Sciences, Daffodil International University, Dhaka, Bangladesh; ^2^ Department of Pathology, Clinical Medical College and The First Affiliated Hospital of Chengdu Medical College, Chengdu, Sichuan, China; ^3^ Institutes for Systems Genetics, Frontiers Science Center for Disease-Related Molecular Network, West China Hospital, Sichuan University, Chengdu, Sichuan, China; ^4^ School of Pharmaceutical Sciences, Lovely Professional University, Phagwara, Punjab, India; ^5^ Department of Chemistry, University of Swabi, Swabi, Pakistan; ^6^ Department of Rasa Shastra and Bhaishajya Kalpana, Faculty of Ayurveda, Institute of Medical Sciences, Banaras Hindu University, Varanasi, Uttar Pradesh, India

**Keywords:** Parkinson’s disease, medicinal plants, natural products, treatment, neurodegeneration, neuroinflammation, pathways

## Abstract

Natural substances originating from plants have long been used to treat neurodegenerative disorders (NDs). Parkinson’s disease (PD) is a ND. The deterioration and subsequent cognitive impairments of the midbrain nigral dopaminergic neurons distinguish by this characteristic. Various pathogenic mechanisms and critical components have been reported, despite the fact that the origin is unknown, such as protein aggregation, iron buildup, mitochondrial dysfunction, neuroinflammation and oxidative stress. Anti-Parkinson drugs like dopamine (DA) agonists, levodopa, carbidopa, monoamine oxidase type B inhibitors and anticholinergics are used to replace DA in the current treatment model. Surgery is advised in cases where drug therapy is ineffective. Unfortunately, the current conventional treatments for PD have a number of harmful side effects and are expensive. As a result, new therapeutic strategies that control the mechanisms that contribute to neuronal death and dysfunction must be addressed. Natural resources have long been a useful source of possible treatments. PD can be treated with a variety of natural therapies made from medicinal herbs, fruits, and vegetables. In addition to their well-known anti-oxidative and anti-inflammatory capabilities, these natural products also play inhibitory roles in iron buildup, protein misfolding, the maintenance of proteasomal breakdown, mitochondrial homeostasis, and other neuroprotective processes. The goal of this research is to systematically characterize the currently available medications for Parkinson’s and their therapeutic effects, which target diverse pathways. Overall, this analysis looks at the kinds of natural things that could be used in the future to treat PD in new ways or as supplements to existing treatments. We looked at the medicinal plants that can be used to treat PD. The use of natural remedies, especially those derived from plants, to treat PD has been on the rise. This article examines the fundamental characteristics of medicinal plants and the bioactive substances found in them that may be utilized to treat PD.

## Graphical Abstract

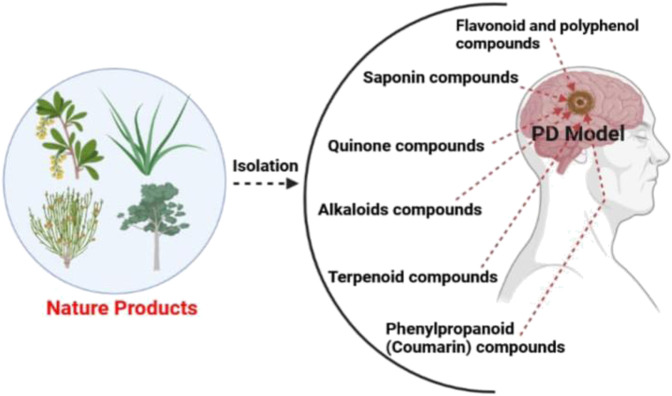

## Highlights

1. Parkinson’s disease (PD) is a chronic progressive neurodegenerative disorder.
2. It can be treated with a variety of natural products and bioactive compounds.
3. Extra care should be provided to patients with PD to minimize the risk of infection.
4. Future perspectives like PD vaccine, cell transplantation, gene therapy, and surgical methods are highlighted.

## 1 Introduction

James Parkinson wrote the first description of Parkinson’s disease (PD) and published it in 1817. PD, also known as idiopathic paralysis agitans, is a frequent ND of the central nervous system (CNS). PD affects about 1% of those over the age of 65. It seldom occurs before the age of 20 and it generally begins between the ages of 40 and 70. Young-onset Parkinson’s disease is a kind of Parkinson’s disease that appears before the age of 20. Parkinsonian symptoms can be observed in other pathologies such as Wilson’s Disease and Huntington’s Disease (Snyder and Wolozin, 2004; Klein and Westenberger, 2012; Postuma et al., 2015). According to the Global Burden of Disease (GBD) survey, there were 1.02 million new cases of Parkinson’s disease in 2017 (Sengupta et al., 2016). Globally, 6.1 million PD patients were recorded in 2016, and the age-standardized prevalence rate (ASR) rose by 21.7% between 1990 and 2016 (Feigin et al., 2016). Years lived with disability (YLDs) is a measure of both the handicap brought on by that status and the average time it takes for incident instances to recover or pass away. YLDs is a popular metric for measuring the health damage brought on by PD. Age-standardized rates of PD-related young life deaths rose sharply from 1990 to 2007, rising 8.9%, and then increased 1.0% from 2007 to 2017 (Global, 2018). According to studies, the burden of PD would significantly increase in the coming decades (Kowal et al., 2013; Wanneveich et al., 2018). For instance, it was predicted that 4.94 million PD patients, or 50% of all PD patients worldwide, will reside in China by 2030 (Dorsey et al., 2007). However, this has not yet been conclusively demonstrated. Variables including overwork, trauma, exposure to cold, rigid personalities and stress are thought to be the predisposing factors (Rahman et al., 2020). Parkinson’s disease is the world’s second most common neurological condition. The etiology of this disease is unknown, but mutations in some genes described in familial PD could help to better understand the role of some proteins and the mechanisms involved in the development of this disorder. PD is triggered by a complex interplay of genetic, environmental, and epigenetic influences. The corresponding risk of developing PD was 2.0% for men and 1.3% for women (ratio = 1.5) (Elbaz et al., 2002; Schneider et al., 2017; Sharma et al., 2018).

Changes in a significant number of other genes were thought to be PD-causative and were discovered by linkage analysis or a candidate gene approach, in addition to the genes responsible for the six monogenic types of PD. Some of these genes—UCHL1 [PARK5], GYGYF2 [PARK11], OMI/HTRA2 [PARK13], PLA2G6 [PARK14], and FBXO7 [PARK15]—even received the “PARKI” designation. A higher risk of developing PD has been linked to variants in a number of PARK-designated (SNCA, UCHL1, LRRK2, PARK 16, GAK) and a few additional genes (MAPT, GBA, NAT2, INOS2A, GAK, HLA-DRA, and APOE) (Postuma et al., 2015). Parkin, DJ-1, ubiquitin C-terminal hydrolase isozyme L1 (UCH-L1), nuclear receptor-related factor 1, and α-synuclein are genes linked to either Parkinson- or Parkinson-related diseases. Because it rapidly aggregates and forms the majority of Lewy bodies (LBs), α-synuclein is particularly noteworthy. The α-synuclein that has accumulated interacts to the proteasome and effectively suppresses proteasomal action. Proteasomal dysfunction is believed to play a role in the pathogenesis of PD because ubiquitin builds up in LBs and interacts with the proteasomal system through interactions with parkin and UCH-L1. Numerous studies suggest that neurotoxins may interact with α-synuclein or other proteins connected to Parkinson’s disease to influence the etiology of the disease (Klein and Westenberger, 2012).

PD is a complex neurodegenerative disorder (ND). LBs and the loss of dopaminergic neurons in the substantia nigra have long been connected to the motor symptoms of Parkinson’s disease (Rahman et al., 2020; Cassotta et al., 2022; Lv et al., 2022). Although oxidative stress, mitochondrial dysfunction, and aberrant protein aggregation have been associated with Parkinson’s disease, the exact origins of the disease remain unclear. Until recently, dopamine replacement therapy was the primary treatment for Parkinson’s disease. Even though many researchers have tried to find a way to stop the neurodegenerative process of Parkinson’s disease, no medicine has been found to protect neurons or change the course of the disease in clinical PD patients. Surprisingly, a wide range of natural chemicals are being used as alternative medicines to treat Parkinson’s disease (Sharma et al., 2018; Li et al., 2019). The most prevalent symptoms of Parkinson’s disease include dyskinesias, muscle tremors, rigidity and anomalies in body posture and movement. Parkinson’s disease causes dopaminergic neurons in the substantia nigra pars compacta to get worse, and the number of transmitters in dopaminergic neurons in the striatum drops by a lot. As a result, nigrostriatal dopaminergic neuron function declines and cholinergic neuron the function rises, resulting in movement problems (Phani et al., 2012).

However, the pathogenic factors discovered in the majority of Parkinson’s disease patients have yet to be verified. The majority of contemporary opinions believe that fibrillation and aberrant α-synuclein aggregation are the primary components in the PD clinical occurrences. Many factors, including oxidative stress and intermediate oligomer conformation, play important roles in the pathogenesis of Parkinson’s disease in different metabolic pathways. Furthermore, the existence of Lewy bodies (LBs) made up of α-synuclein is a significant pathological marker of PD. As a result, α-synuclein may be linked to PD (Stefanis, 2016; Zhang et al., 2017a; Zhang et al., 2017b; Sharma et al., 2019a; Rahman et al., 2022a). Furthermore, a growing body of evidence suggests that many common molecular signaling pathways are linked to the development and progression of Parkinson’s disease, thanks to recent and updated developments in life sciences technologies, as well as ongoing in-depth research on proteomics and molecular biology. The phosphoinositide 3-kinase/protein kinase B pathway is the most important of them (Nakaso et al., 2008; Quesada et al., 2008), the nuclear factor erythriod2-related factor2 (Williamson, 2012; Wang et al., 2014), the P38 mitogen activated protein kinase (Wilms et al., 2003; Corrêa and Eales, 2012), the glycogen synthase kinase-3b (Wu et al., 2007; Duka et al., 2009; Golpich et al., 2015), the c-jun-N-terminal kinase (Hunot et al., 2004; Kuan et al., 2005), the nuclear transcription factor-κB (NF-κB) (Uberti et al., 2004; Noda et al., 2005), the Want signaling pathway (Berwick and Harvey, 2012; Arenas, 2014), and the autophagy lysosome pathway (Xilouri et al., 2008; Xilouri and Stefanis, 2011). This article discusses newly identified natural compounds having substantial anti-PD capabilities, as well as the current state of research into their medicinal chemistry.

## 2 Etiology of PD

In the past, the cause was completely random, but now both environmental and genetic factors are thought to play a role (Schapira, 2006; Schapira and Tolosa, 2010; Schapira, 2011), and understanding of etiological elements in Parkinson’s disease has altered substantially. However, age or the aging process is the single most important predictor of the beginning of PD. Despite the certainty of age’s significance, little effort appears to have been made to investigate how age and the aging process interact (Obeso et al., 2010; Sharma and Martins, 2020; Sharma and Prajapati, 2020). The most widespread explanation is that as normal cellular physiological and biochemical processes fail more frequently, dopaminergic neurons become more sensitive to toxic assault. Dopaminergic cells aging linked with L-type calcium channels (Surmeier et al., 2010) is a recent example. In reality, most models of cell death in PD disregard the role of age or aging, and young animals are used to represent the disease process in the great majority of experimental research (Hindle and Ageing, 2010). When looking at the prevalence of PD, the same might be said about men outnumbering women (Shulman, 2007). This must also provide insight into the etiology of the illness, especially given men’s shorter life expectancy. Despite evidence that estrogen plays a role in determining the age at which women develop PD and its effect on dopaminergic neuron function, no explanation for the disparity has emerged. Industrialization, rural areas, well water, plant-derived toxins, bacterial and viral infection, and exposure to organic solvents, carbon monoxide and carbon disulfide are some of the broad environmental factors on the prevalence of PD (Tellier et al., 2022). Despite inconsistent results from various research and the difficulty of identifying specific pesticide compounds that may be connected to an increased risk of PD, pesticide exposure has recently attracted interest (Richardson et al., 2009). Paraquat and rotenone are two specific agrochemicals that have been shown to cause dopaminergic cell death in the nigral area of mice (Berry et al., 2010; Chia et al., 2020). Some of these will be discussed later while discussing pathogenic mechanisms. Factors that reduce the risk of developing Parkinson’s disease can also provide important information about the disease’s progression. Exercise, anti-inflammatories, antihypertensives (mostly calcium antagonists) and antilipidemic all seem to lower the risk, but the role of some, like anti-inflammatories, exercise, antihypertensives (mostly calcium antagonists) and antilipidemic, is still unknown (Ascherio et al., 2001; Warner and Schapira, 2003). Even though there are many different factors that have been linked to the pathogenesis of Parkinson’s disease, it was the discovery of the neurotoxic effects of MPTP that got the most attention and started a new era of research (Burns et al., 1983; Langston et al., 1983; Jenner et al., 1984). When MPTP was found, it led to research into how it works and how it might be related to the cause of Parkinson’s disease. But perhaps more importantly, it gave us the first model of Parkinson’s disease motor deficits in primates and a predicted test bed for therapeutic action in people (Jenner, 2003). However, MPTP-induced parkinsonism differs from typical PD in that it does not develop in humans, there is no LB development and there is no pathology in other parts of the brain that are damaged in human illness. As a result, despite its significance, MPTP may fail to send the critical information required to answer the genesis of the Parkinson’s disease enigma (Noyce et al., 2016).

## 3 Pathogenesis of PD

PD is a neurological, progressive condition that causes a number of motor and crippling abnormalities, including as bradykinesia, resting tremor, muscle stiffness and imbalance. Dopamine levels in the striatum, tailed nuclei and putamen are reduced as a result of the slow, progressive degeneration of dopaminergic neurons in the SN compacta, which is a pathological hallmark of PD. The most significant pathogenic finding in the brains of PD patients is the progressive loss of dopaminergic neurons in the basal complexes. Dopamine neurotransmitter levels in this region are decreased as a result of the destruction of these neurons. The disease’s symptoms start to show up when 50%–60% of dopaminergic neurons have been destroyed and dopamine levels in the striatum have dropped by about 80%–85%. However, studies have shown that oxidative stress and mitochondrial dysfunction probably play a key role in the pathogenesis of PD; the loss of nigrostriatal dopaminergic neurons and the presence of intracellular cytoplasmic proteins, i.e., Lewy bodies, are also involved. The precise molecular mechanism of the degradation of dopaminergic neurons and the incidence of PD is unknown. The cells are transmitted to the putamen from the nigrostriatal neurons in the SN pars compacta (SNpc). Depigmentation of SNpc results from the absence of these neurons, which normally contain modest levels of melanin (Lees, 2012). PD, the most prevalent form of Multiple Sclerosis (MS), results from the progressive degeneration of dopaminergic neurons along the nigrostriatal pathway (Hacker et al., 2012; Luo et al., 2014; Prodoehl et al., 2014). Magnetic resonance diffusion tensor imaging revealed signs of neurodegeneration in the nigrostriatal circuits (Wang et al., 2015). Early-onset Parkinson’s disease was shown to have frontal and parietal lobe microstructural damage, which was connected to postural and walking issues (Gattellaro et al., 2009; Gu et al., 2014; Auning et al., 2015). Furthermore, radial alterations in nigrostriatal fibers have been associated with the degree of movement impairments in Parkinson’s disease patients (Zhang et al., 2015). PD is caused by a variety of causes that cause neurodegeneration.

### 3.1 Mitochondrial dysfunction in PD

Recently some researcher found missing of the substantial nigra of the PD patients, investigators also revealed abnormalities of the mitochondria in skeletal muscle, platelets and lymphoblast that basically indicate direct link between mitochondrial dysfunction and PD (Schapira et al., 1989; Schapira et al., 1990; Schapira, 1994). PD patients with mitochondrial dysfunction confirmed by pathologically especially in sporadic PD, against a backdrop of increased oxidative stress and elevated brain iron levels, this helps us to understand how mitochondrial abnormalities interconnected in PD pathogenesis (Dexter et al., 1992; Mann et al., 1994; Olanow et al., 1995; Schapira, 1995; Schapira, 2011). The discovery of specific mutations in genes that cause dopaminergic cell death and familial PD have confirmed the role of mitochondria in the development and pathophysiology of PD. Many instances of PD with PINK1 or LRRK2 mutations, on the other hand, are clinically indistinguishable from spontaneous PD. Several knockouts or expression models of these mutations have been discovered to exhibit mitochondrial function abnormalities (Moon, 2007). Parkin is a ubiquitously transcribed protein that has been linked to the endoplasmic reticulum, synaptic vesicles, Golgi apparatus, and mitochondria in intracellular investigations (Shimura et al., 1999; Kubo et al., 2001; Mouatt-Prigent et al., 2004).

### 3.2 LBs and microgliosis

The alternative method to investigating PD etiology has been to look at the pathological components of the disease process. The formation of LBs and the existence of a reactive microgliosis, which may contribute to disease progression, are two characteristics of PD. The LB has long been regarded as the distinguishing hallmark of PD, although its significance in the disease process has been debated. It has been alternately described as a pathogenesis marker and a graveyard for dead and dying neurons. In PD α-synuclein mutations is common and in sporadic PD LBs strongly immunoreactive for wild-type α-synuclein with many other protein in their normal or damaged condition (Spillantini et al., 1998). The discovery of parkin and UCH-L1 mutations, as well as their involvement in the ubiquitin-proteasomal system, ushered in a new age of research into changes in protein processing in dopaminergic cells *via* both proteasomes and lysosomes (Eldeeb et al., 2022; Kumar et al., 2022). The argument has been fanned by postmortem tests and toxicology research. In the substantia nigra, there is a decrease in proteasomal enzyme activity that is unique to PD and does not occur in other parts of the brain (McNaught et al., 2003; Tofaris et al., 2003).

### 3.3 Neuroinflammation in PD

**FIGURE 1:**
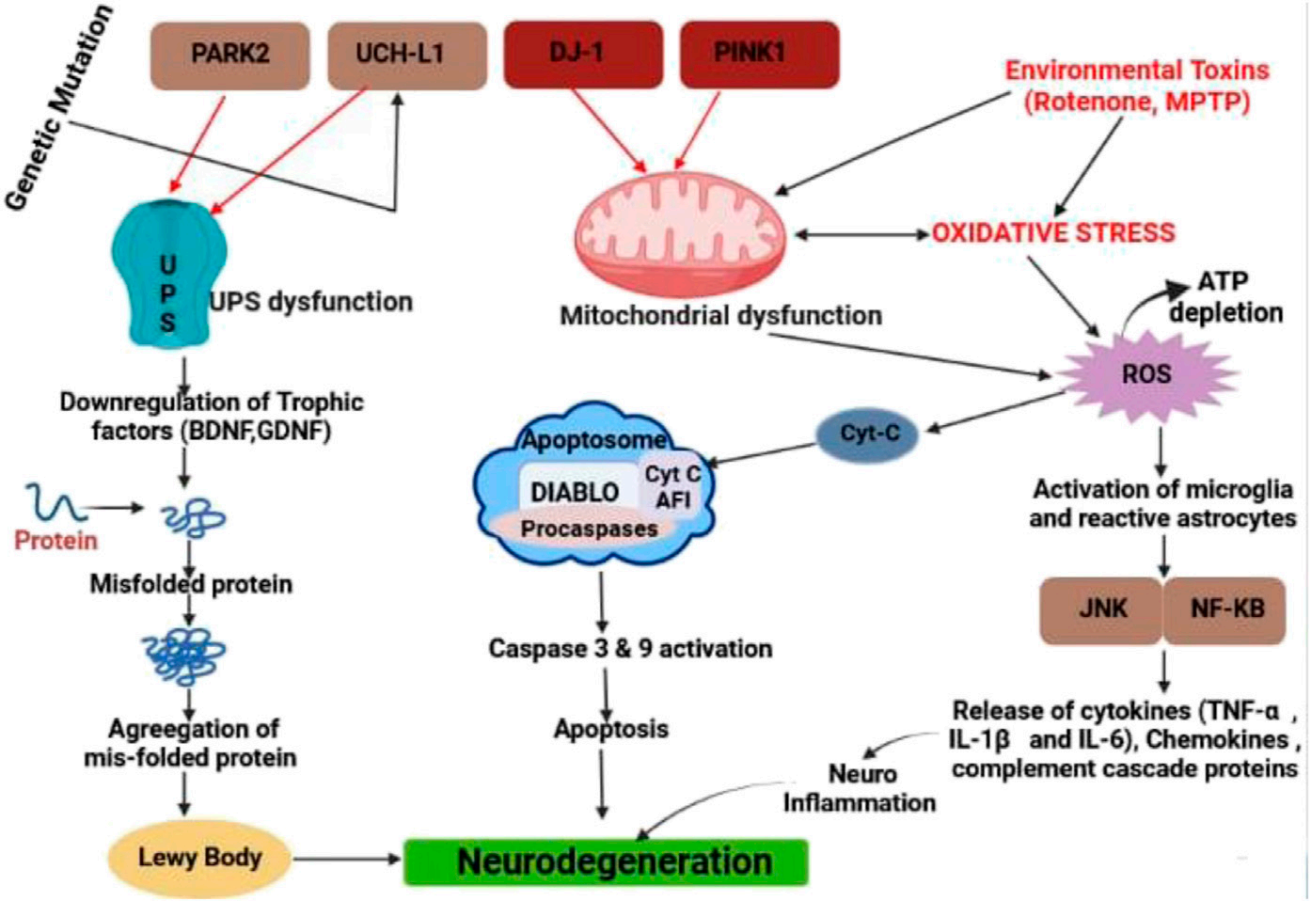
Different types of pathway for neurodegeneration in PD by LB, caspase activation and releasing different types of inflammatory cytokine.

McGeer et al. discovered that post-mortem brains of people with PD exhibit elevated human leucocyte antigen, which was the first indication of neuroinflammation’s role in PD pathogenesis in 1988. Microglia with DR positivity (McGeer et al., 1988). Furthermore, elevated pro-inflammatory mediators such as TNF, ILβ, IL6, iNOS, and COX two have been found in the striatum and substantia nigra, according to this study (Tansey et al., 2007). Microglia are one of the most important kinds of cells in the central nervous system’s inflammatory response (Saijo et al., 2009). As previously mentioned, PD is linked to aberrant α-synuclein aggregation, which activates microglia even more (Su et al., 2008). Neuroinflammation is characterized by the activation of microglia and reactive astrocytes in the brain, as well as the production of inflammatory mediators such as cytokines (TNF-α, IL-1, and IL-6), chemokines, complement cascade proteins, reactive oxygen species, and reactive nitrogen species (RNS). Several factors have been shown to reduce the permeability of the blood-brain barrier (BBB) (da Fonseca et al., 2014) (Figure 1). The degeneration of the nigral dopaminergic neurons in PD is caused mainly by inflammasome-induced neuroinflammation. Baicalein, a flavonoid derived from the *Scutellaria baicalensis Georgi* plant used in traditional Chinese medicine, has been shown to have anti-inflammatory and neuroprotective effects in animal models of NDs, including PD. In a PD mice model induced by MPTP, baicalein inhibits the NLRP3/Caspase-1/GSDMD Pathway to decrease neuroinflammation (Rui et al., 2020).

## 4 Role of natural products

### 4.1 Herbal products

#### 4.1.1 Baicalein

Baicalein is a chemical compound derived from the dried root of the *Scutellaria baicalensis* plant (Labiatae) (Amro et al., 2018). Baicalein prevented the buildup of ROS, apoptosis, ATP depletion and mitochondrial membrane rupture in PC12 cells when tested for rotenone-induced neurotoxicity (Li et al., 2012). Baicalein treatment prevents Dopamine levels in the basal ganglia from dropping and boosts Dopamine and 5-hydroxytryptamine levels (Cheng et al., 2008; Mu et al., 2011). In Hela and SH-SY5Y cells, Baicalein inhibited the aggregation of α-synuclein and the production of α-synuclein oligomers (Lu et al., 2011).

#### 4.1.2 Erythrina velutina

The ethanol extract of this plant (Fabaceae) has a neuroprotective effect. It has been shown to reduce the neurotoxicity caused by 6-OHDA in SH-SY5Y cells and to get rid of free radicals, which suggests it could be used to treat Parkinson’s disease (Silva et al., 2016).

#### 4.1.3 Resveratrol

Resveratrol is a polyphenolic compound present in a variety of plants, including grapes and berries (Frémont, 2000; Rahman et al., 2021a). Resveratrol has been found to help with motor deficits, oxidative stress, and the loss of TH neurons in animal models of Parkinson Disease (Lu et al., 2008). Resveratrol inhibits mitochondrial enlargement and chromatin condensation while also lowering COX-2 and TNF-α gene expression (Jin et al., 2008).

#### 4.1.4 Peganum harmala

*Peganum harmala* (Nitrariaceae) made muscles less stiff, stopped oxidation of fats and proteins in the brain and stopped dopaminergic neurons from dying off (Rezaei et al., 2016). It is thought that this herb’s neuroprotective properties come from its ability to reduce the activity of angiotensin II. This reduces oxidative stress and protects dopaminergic neurons (LopezReal et al., 2005).

#### 4.1.5 Curcuma longa

*Curcuma longa* (Zingiberaceae) has been shown to have anti-inflammatory, chemotherapeutic, anti-oxidant, wound-healing, anti-proliferative and antiparasitic properties. The active component polyphenolic fraction, curcumin, is probably to blame (Gupta et al., 2012). Curcumin protects MPTP-induced loss of TH-positive neurons and DA depletion in the striatum of MPTP-induced mouse models, as well as a reduction in cytokines, total nitrite, and inflammatory markers such inducible nitric oxide synthase (Ojha et al., 2012).

#### 4.1.6 *Carthamus tinctorius* L. (Safflower)

Safflower (Asteraceae) has been discovered to contain flavonoids and is widely used as a conventional treatment for cerebrovascular disorders in China (Amro et al., 2018). It increased DA transporter and DJ-1 protein expression as well as DA levels (Ren et al., 2016). Overexpression or aggregation of α-synuclein, as well as reactive astrogliosis, may be inhibited by safflower (Ren et al., 2016).

#### 4.1.7 Pueraria lobata

Puerarin (Fabaceae) has been shown to inhibit proteasomal malfunction as well as the buildup of ubiquitin-conjugated proteins and other potentially hazardous proteins (Amro et al., 2018). On the other hand Puerarin, lowers the ratio of bcl-2/bax and caspase-3 activity (Cheng et al., 2009). Puerarin protects tyrosine hydroxylase (TH)-positive neurons from 6-OHDA-mediated injury, recovers DA and its metabolites (Zhu et al., 2010).

#### 4.1.8 Juglandis semen

The neuroprotective effects of aqueous *Juglandis semen* (walnut) extract have been demonstrated. The walnut extract was reported to reduce reactive oxygen species (ROS) and nitric oxide (NO) formation as well as restrict the depletion of striatal DA and its metabolites, resulting in a considerable improvement in PD movement abnormalities in a mouse model of Parkinson’s disease (Choi et al., 2016). Walnut is thought to have neuroprotective effects because it can block the monoamine oxidase B (MAO-B) enzyme, which increases oxidative stress in people with Parkinson’s disease. Walnut also has antioxidant and mitochondrial protection properties (Essa et al., 2015).

#### 4.1.9 Ginkgo biloba

*Ginkgo biloba* (Ginkgoaceae) is a Chinese tree that has long been used to treat symptoms related to heart and lung problems. Flavonoids, ginkgolic acid and terpenoids are three of the most common constituents in *G. Biloba* (DeFeudis and Drieu, 2000). In a PD rat model treated with 1-methyl-4-phenyl-1,2,3,6-tetrahydropyridine (MPTP), long-term use of EGb761 prevented the loss of dopaminergic nerve terminals caused by MPTP (Ramassamy et al., 1990). EGb761 was shown to protect against dopaminergic neurotoxicity caused by MPTP whether it was given before or after the treatment (Sharma et al., 2019b). Also, EGb761 decreased the neurotoxicity of levodopa in the 6-hydroxydopamine (6-OHDA) Parkinson’s disease (PD) model. This suggests that levodopa is neurotoxic and that EGb761 may reduce this toxicity (Fei et al., 2003).

#### 4.1.10 Ginseng

Ginsenosides (Araliaceae) Rb1 and Rg1 are regarded to be the primary molecules responsible for ginseng’s therapeutic properties. A previous study found that the ginsenosides Rb1 and Rg1 both decreased MPTP-induced cell death in SN-K-SH cells (a neuroblastoma cell line) (Rudakewich et al., 2001). Rg1 protects cells against apoptosis caused by MPTP by increasing Bcl-2 and Bcl-xl expression, decreasing Bax and iNOS expression and blocking caspase-3 activation (Chen et al., 2002). Ginsenosides protect by lowering intracellular reactive oxygen species (ROS), boosting antioxidant activity, maintaining complex I activity, and raising intracellular Adenosine triphosphate **(**ATP) levels, according to research. Mice given 1-methyl-4-phenyl-1,2,3,6-tetrahydropyridine (MPTP) had better motor function and more dopaminergic neurons in the substantia nigra (SN) and striatum when they were given Rg1 (Jiang et al., 2015). In addition, the ginsenoside Rb1 has the ability to disaggregate fibrils and inhibit α-synuclein polymerization (Ardah et al., 2015; Rahman et al., 2021b).

#### 4.1.11 Flavonoids

Flavonoids are a type of natural polyphenol phytochemical that has been used as a medicinal agent for many years. Baicalin, a flavonoid derived from *Scutellaria baicalensis*, is the major metabolite of baicalein (Lamiaceae). In an *in vivo* model of Parkinson’s disease with the neurotoxin 6-OHDA, which showed protective effects on dopaminergic dysfunction and lipid peroxidation, the neuroprotective effects of baicalein were shown to be real (Im et al., 2005). Lutein and apigenin, two flavones, protect dopaminergic neurons against inflammatory neurotoxicity. (Chen et al., 2008).

#### 4.1.12 Valeriana officinalis

*Valeriana officinalis* (Valerian) is a sedative and antispasmodic herb that has long been used for sleeplessness, anxiety, and restlessness. In SH-SY5Y cells, valerian has been shown to inhibit rotenone-induced cell death (Amaral de Brito et al., 2020). Furthermore, valerian extract was effective in reducing rotenone toxicity in *Drosophila melanogaster*, as evidenced by the normalization of superoxide dismutases-SOD and catalase mRNA expression, implying that valerian’s effects are, at least in part, due to the plant’s antioxidant properties due to its phenolic and flavonoid constituents (Sudati et al., 2013).

#### 4.1.13 Passion flower

Flavonoids, glycosides, alkaloids, and phenolic chemicals are all found in passion flowers, also known as *Passiflora incarnata* (Passifloraceae). Anxiety, epilepsy, sleeplessness, muscle spasms, and other disorders have all been treated with it (Dhawan et al., 2004). As a result, the biological effects of passion flowers on people with Parkinson’s disease have been investigated. The quantity of jaw movements produced by tacrine, a typical animal model of Parkinson’s disease tremors, was reduced by passionflower extract. The animal’s cognitive abilities improved as well, with haloperidol-induced catalepsy lasting significantly less time. As evidenced by its strong scavenging ability, the passionflower has antioxidant activity (Ingale and Kasture, 2017).

#### 4.1.14 St. John’s Wort

St. John’s wort (Hypericaceae) contains naphthodianthrones, phloroglucinols, flavonoids as well as essential oils, have all been found as active ingredients. As a result, the active ingredients have antioxidative and neuroprotective properties (Barnes et al., 2001). We looked at how two standardized extracts of St. John’s Wort affected neurodegeneration in rats caused by long-term rotenone treatment. St. John’s wort, through lowering Bax levels, decreased neuronal damage and prevented the apoptotic process (Kiasalari et al., 2016). Furthermore, rats with intrastriatal 6-hydroxydopamine (6-OHDA) lesions were given a St. John’s wort extract, which resulted in lower striatal malondialdehyde levels, increased catalase activity, decreased glutathione (GSH) content, normalized Glial fibrillary acidic protein (GFAP) and tumor necrosis factor alpha or TNF-α expression, reduced Deoxyribonucleic acid (DNA) fragmentation, and prevented dopaminergic neuron damage (Kiasalari et al., 2016).

#### 4.1.15 Terpenoids

*Centella asiatica* is a medicinal herb that appears to help with rheumatoid arthritis, inflammation, and mental and physical weariness. Bornyl acetate, α**-**pinene, β-pinene and δ-terpinene are all monoterpenes found in the leaves of this plant (Asakawa et al., 1982; Brinkhaus et al., 2000). Acetylcholinesterase (AChE) activity was shown to be inhibited by these monoterpenes. *C. paniculatus* ethanolic plant extract exhibited sedative and antidepressant properties (*in vivo*) (Sakina and Dandiya, 1990). Compound 5 reduced Ab formation in neuroblastoma cells transfected with amyloid precursor protein (APP) constructs. Toxiigenin also reduced the activity of b-proteolytic secretase on its substrate. In the same way, a pharmacological study found that *P. tenuifolia* (BT-11) dried root extract (80% ethanol-water) could help rats with scopolamine-induced amnesia learn and remember better (Jia et al., 2004).

#### 4.1.16 Phenolics

*Curcuma longa* has been used for anti-aging in Ayurvedic medicine for thousands of years. Curcumin, an antioxidant and anti-inflammatory substance, is found in the rhizomes of *Curcuma longa* (Arshad et al., 2017; Shah et al., 2021; Memarzia et al., 2022; Salem et al., 2022). The compound also showed neuroprotective properties when it came to ethanol-induced brain damage. Curcumin reduced lipid peroxide levels while increasing glutathione levels when taken orally (Kim et al., 2001). Curcumin, demathoxycurcumin, bis-demethoxycumin, and calebin-A, all extracted from Curcuma longa, have been shown to protect PC12 cells from β-amyloid assault (Maurer et al., 1997; Park and Kim, 2002). The stem bark of *Knema laurina* was used to isolate compounds 18–22. The most significant Acetylcholinesterase (AChE inhibitory activity was found in compound 22, which had an IC_50_ of 0.573 mM (Akhtar et al., 2011). *In vitro* experiments showed that a fruit extract from Styraxagrestis suppressed AChE. Three novel egonoltypebenzofurans were isolated using a bioassay-guided fractionation and isolation method: egonol-9(Z),12(Z) linoleate, 7-demethoxyegonol9(Z),12(Z) linoleate and 7-demethoxyegonol oleate (IC50 = 1.4–3.1 mM) (Liu et al., 2011).

#### 4.1.17 Alkaloids

*In vitro* and *in vivo*, the lycopodium alkaloid huperzine A (structurally similar to quinolizidine) obtained from *H. serrata* is a reversal inhibitor of AChE (Small et al., 1997; Shu, 1998; Wang et al., 2000; Zhou et al., 2001). It stopped acetylcholinesterase from working (IC_50_ = 0.08 mM) (Xiao et al., 2002). The alkaloid leonurine, derived from *Leonurus heterophyllus* Sweet, inhibited ROS, protected mitochondrial integrity, and reduced cytochrome c levels *in vivo* (Kuribara et al., 2000). The rhizomes of *Coptis chinensis* have yielded numerous alkaloids, including berberine, groenlandicine, palmatine, jateorrhizine, coptisine, and epiberberine. These compounds can stop AChE from doing its job. In addition, the compounds groenlandicine and epiberberine inhibited beta-secretase enzymatic activity (Karakida et al., 2007). The marine *Streptomyces* sp. Strain LB173 produced a novel phenazine natural product called geranylphenazinediol. With an IC_50_ value of 2.62mM, compound 91 inhibited the AChE enzyme (Ohlendorf et al., 2012).

#### 4.1.18 Mucuna pruriens

*Mucuna pruriens* (Mp) has therapeutic qualities in all of its components. They are said to have anti-inflammatory, analgesic, anti-epileptic, anti-neoplastic and anti-microbial properties (Adepoju and Odubena, 2009). They showed that Mp is a better way to treat Parkinson’s disease over the long term than standard L-DOPA therapy, which causes severe dyskinesia when used for a long time. MP’s seed, leaf, and stem all have significant neuroprotective properties. Because seeds have more L-DOPA than other parts of the plant, they are often used as anti-PD drugs (Benfica et al., 2020).

#### 4.1.19 Withania somnifera

Environmental poisons Maneb (MB) and paraquat (PQ) have been used in tests to generate selective damage to dopaminergic neurons, leading to the development of Parkinson’s disease (PD). When an ethanolic root extract of *Withania somnifera* (Ws) was given to a mouse model of Parkinson’s disease caused by MB-PQ, it was shown to significantly improve classic Parkinson’s disease symptoms like slow movement, less dopamine in the substantia nigra and different types of oxidative damage (Ahmad et al., 2005; Yadav et al., 2012).

#### 4.1.20 Tinospora cordifolia

*T. cordifolia* is classified as “Rasayana” in Ayurveda and is used to combat infections due to its general adaptogenic and prohost immunomodulatory effect. It has antiandrogenic and anti-inflammatory properties, is effective against throat cancer, stress, and promotes learning and memory. In an acute toxicity investigation, *T. cordifolia* was shown to be nontoxic. It has recently been intensively researched and shown to have significant antioxidant action. Because antioxidants are known to prevent or protect neurodegeneration, the current study was designed to assess the anti-Parkinson’s efficacy of *T. cordifolia* ethanol extract (TCEE) (Kosaraju et al., 2014).

#### 4.1.21 Bacopa monnieri

*In vitro* and in animal models of neurodegenerative illness, *B. monnieri’s* antiparkinsonian efficacy has been studied *in vitro*. *B. monnieri’s* antioxidant and neuroprotective properties confer anti-Parkinsonian action, which is related to decreased-synuclein protein aggregation and the selective death of dopaminergic neurons. In worms, *B. monnieri* reduced synuclein aggregation, prevented dopaminergic neurodegeneration, and restored lipid content, indicating that this species may have anti-Parkinsonian effects (Hosamani, 2009).

### 4.2 Marine derived compound

Natural marine-derived chemicals might have several pharmacologic effects and could be extremely beneficial for the creation of novel medicines (Corona, 2018).

#### 4.2.1 Archaea

Many superheats may produce Zwitterionic organic products to avoid thermal denaturation and protein aggregation under severe circumstances like as extreme temps and osmolarity (Empadinhas and da Costa, 2011). In a hot environment, hyper thermophilic bacteria produce mannosylglycerate (MG) as a suitable solute. In a PD yeast model, MG inhibited the development of α-synuclein aggregates. Mannosylglycerate may help α-synuclein fold properly, preventing abnormal aggregation. MG is a promising therapy for PD (Faria et al., 2013).

#### 4.2.2 Bacteria

Ancillary marine compounds provide a rich pharmacological source with new chemical structures and a wide range of biological activity (Nikapitiya, 2012; Monciardini et al., 2014). NP7 is a marine *Streptomyces* sp. Compound (NP7) is an anti-oxidant and could cross The BBB. H2O2, caused Apoptosis and H2O2 in nerves and Microglia cells NP7 at 5–10 μM might avoid (Koppula et al., 2012). Piloquinones, marine-derived chemicals isolated from *Streptomyces* sp. Have been found to have inhibitory action of MAO-B (Takeuchi et al., 1973). The *Streptomyces* sp. CNQ-027 isolated piloquinones A, and B (Nam et al., 2011).

#### 4.2.3 Fungi

Many marine fungus metabolites can have neuroprotective anti-PD actions. Neoechinulin A is an isoprene quinone alkaloid produced by the reddish algae-based fungi Aspergillus sp. and Microsporum sp (Li et al., 2004). Neoechinulin A might protect PC12 cells from the neuronal mortality of MPP+ and peroxynitrite through the reversal of mitochondrial complex I malfunction (Kimoto et al., 2007; Kajimura et al., 2008). The natural substance is *Aspergillus ochraceous* and Paecilomyces sp, secalonic acid A derived from marine fungal (Kurobane et al., 1987). The suppression of p38 phosphorylation and JNK and reducing Calcium entry and caspase-3 activation have dramatically reduced the induced colchicine apoptosis of cortical nerve cells at 3–10 μM (Zhai et al., 2011).

#### 4.2.4 Algae

Marine algae have a strong antioxidant supply (Meenakshi et al., 2011). Carotenoids, notably astaxanthin, produced by marine micro-organisms, have proved to be a useful adjuvant therapy in preventing and/or delays of NDs, for example, *Haematococcus pluvialis* and *Chlorella zophingiensis* (Yuan et al., 2011; Galasso et al., 2017). Astaxanthin may generate mouse anti-PD effects (Grimmig et al., 2017a). Astaxanthin has been proven to decrease microglial activation in the mouse brain (Grimmig et al., 2017b). The marine carotenoid of Fucoxanthin that comes from edible brown seaweeds has demonstrated antioxidant and anti-inflammatory effects (Gammone et al., 2015). By stimulating the PI3-K/Akt cascade, Fukoxanthin might reduce H_2_O_2_ neuro substances and block the ERK route (Lin et al., 2017).

#### 4.2.5 Mollusk

A kinase inhibitor called staurosporine (AM-2282) was first discovered in the actinomycete *Streptomyces staurosporeus* (Karaman et al., 2008). Additionally, flatworm and marine sea squirt contained staurosporine (AM-2282) (Schupp et al., 2001). Staurosporine (AM-2282) at 10 nM might promote DA neurite outgrowth by activating the AMP-activated protein kinase (AMPK)/mammalian target of rapamycin (mTOR) signaling pathway in mesencephalic primary cultures (Wakita et al., 2014). Additionally, staurosporine (AM-2282) may shield neurons from damage brought on by ischemia (Hara et al., 1990). Staurosporine is extremely poisonous, nevertheless. Staurosporine analogues have been generated by structural alteration to reduce toxicity (Bharate et al., 2013).

#### 4.2.6 Sea cucumber

A marine mollusc called the sea cucumber has vital nutrients. In many Eastern nations, sea cucumber is recognized as a tonic and a traditional therapy for neurodegenerative illnesses. Whole body-ethyl acetate (WBEA), whole body-butanol (WBBU), and body wall-ethyl acetate are extracts of the sea cucumber *Holothuria scabra* (BWEA). These compounds can stop the loss of DA neurons caused by 6-OHDA in *Caenorhabditis elegans* by a large amount. Additionally, these extracts could restore lipid content and prevent the aberrant aggregation of α-synuclein (Chalorak et al., 2018). SCG-1, SCG-2 and SCG-3 are significant sphingolipids found in *Cucumaria frondosa*’s sea cucumber extract (Xu et al., 2013). SCG-1, SCG-2 and SCG-3 may promote neurite outgrowth in NGF-induced PC12 cells in a dose-dependent and structure-specific manner, most likely through boosting TrkA phosphorylation and upregulating BDNF expression (Wang et al., 2018). These findings showed that the active components in sea cucumber extracts and their potential anti-PD capabilities.

## 5 Disorder associated with PD

**FIGURE 2:**
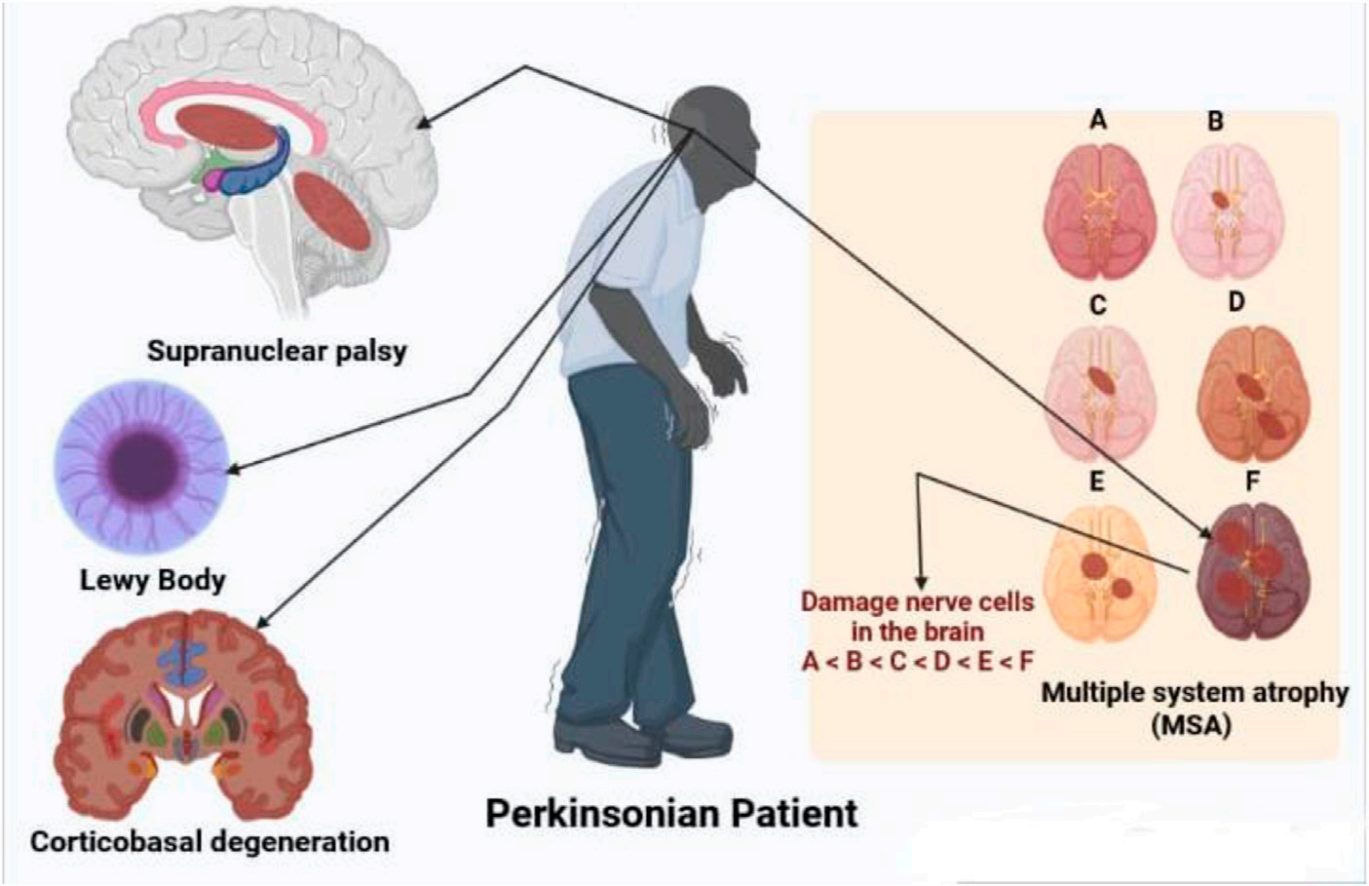
Disorders associated with parkinsonian patient like supranuclear palsy, LB multiple system atrophy and corticobasal degeneration.

Some disorders associated with PD are shown in Figure 2.

### 5.1 Supranuclear palsy

PSP and CBD are the two most frequent tauopathic PD. PSP affects 6.4 persons out of 100,000 (Ikram et al., 2021). Initiating age is an average of 63, males are most affected and survival time is 6–7 years (Wenning et al., 1998). PSP is defined by the early onset of widespread and stiff reverse dips, as well as supranuclear vision paralysis with slow relative direction and difficulty staring down (Litvan et al., 1996a). The NINDS-SPSP clinical diagnosis for probable PSP are based on vertically supranuclear vision paralysis and noticeable poor balance, which occur within the first year of illness start and have the high selectivity (50–62)%, specificity 100%, and positively useful predictions (Litvan et al., 1996b; Litvan et al., 2003).

### 5.2 Multiple system atrophy

Sporadic progressive adult diseases, with an incidence of around 4.4 instances per 100,000 population, are multiple system atrophies (Schrag et al., 1999; Watanabe et al., 2002). The existence of at least six of the following functions have been consistently identified for MSA: Sporadic adult-onset, self-representation, parkinsonism, cerebral characteristics, pyramidal symptoms, there is a shortage of levodopa, downward ocular paralysis and cognitive dysfunctions (Litvan et al., 1998). Diagnosis of MSA is missed even at tertiary reference centers, in 50%–75% of cases (Litvan et al., 1997). Nigral and putamen degeneration and degeneration in at least one region are part of pathological diagnostic criteria (Ito et al., 1996).

### 5.3 Dementia with LBs or LB disease

The second most frequent kind of dementia in advanced age is progressive dementia with LBs, typically accompanied by parkinsonism, good visual hallucinations, and oscillations in cognition, alertness, and concentration (McKeith et al., 1996). DLB is dementia that affects the optical, perceptual, and careful functions of the brain (Collerton et al., 2003). The age at which the DLB begins is 60–68, with an average disease duration of 6–7. Men are more impacted than women (Gualtieri, 2004). DLB diagnostic criteria differ substantially in their sensitivity and specificity and improved criteria are needed (Lopez et al., 2000). Cases with more substantial DLB disease have typical symptoms, whereas cases with larger neurofibrillary tangles are likely to show AD (Ballard et al., 2004). Continuing to work memory issues, visual spatial difficulties, psychotic episodes, melancholy, unconcern, and low mood are among early signs of DLB (Simard et al., 2000).

### 5.4 Corticobasal degeneration or degeneration of corticobasal ganglions

Degeneration of the corticobasal ganglion is gradual, with unilaterally akinesia and rigidity responding badly to apraxia (especially ideomotor apraxia) and levodopa. Myoclonus of the cerebral reflex, limb rigidity, alien limb signs, and cortical sensory loss is all symptoms (Riley et al., 1990; Stover and Watts, 2001). Despite the fact that there are various requirements for diagnoses were submitted, none were validated and their warnings were explored elsewhere (Litvan et al., 2003). The estimated prevalence per 100,000 individuals is 4.9–7.3 cases (Togasaki and Tanner, 2000).

### 5.5 PD and sleep disorders

#### 5.5.1 Recurrent PD symptoms and sleep fragmentation

**FIGURE 3:**
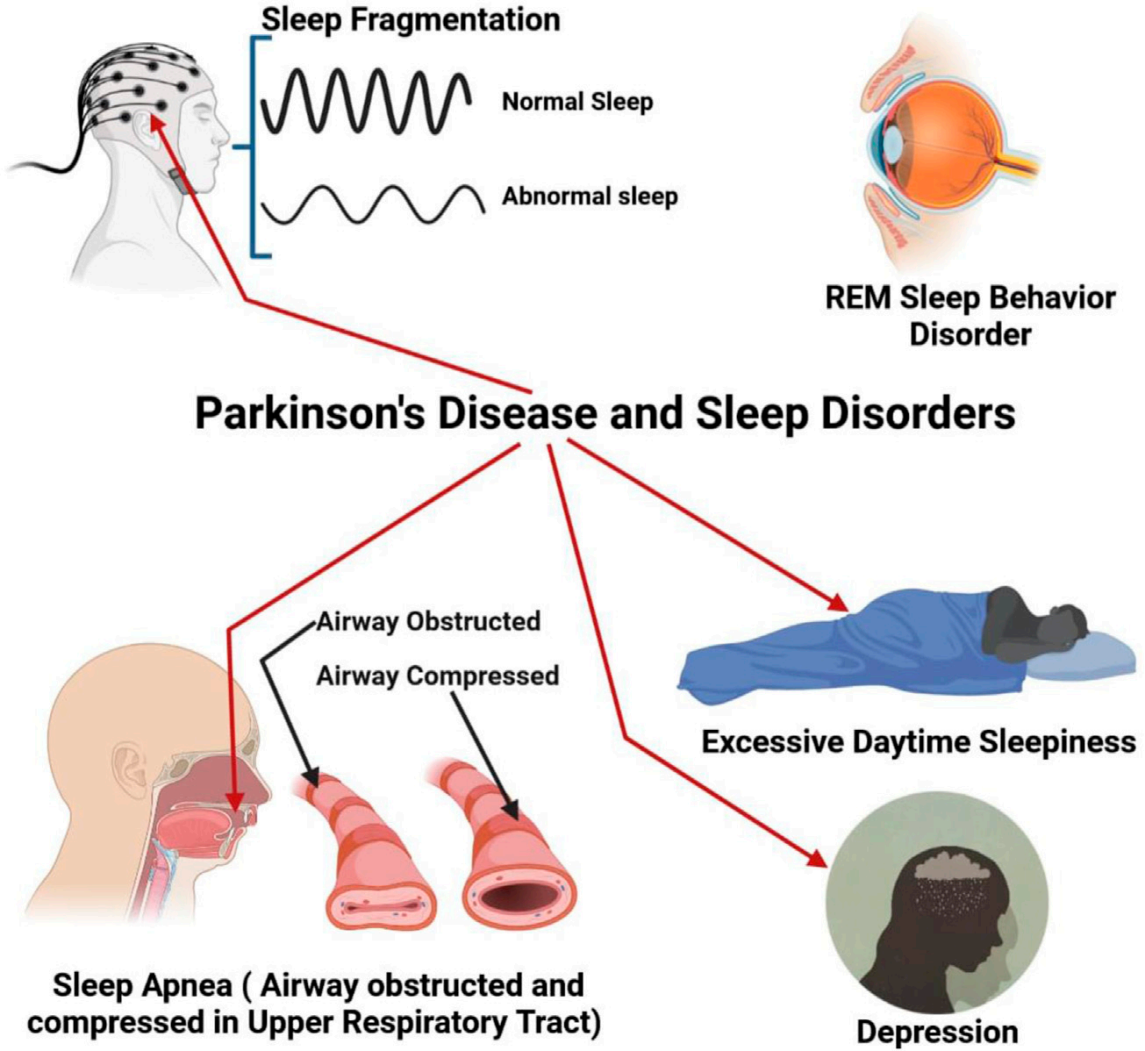
PD with different types of sleep disorder like sleep fragmentation, REM (Rapid Eye Movement), Breathing problem during sleep (Apnea), increase daytime sleep and finally depression.

Insomnia at night and daytime sleepiness are prevalent among people with PD (Figure 3). 60 to 98 percent of PD patients experience nighttime difficulties (Kales et al., 1971; Lees et al., 1988; Larsen, 2003). 33% of individuals experience moderate to severe sleep difficulties at night (Svensson et al., 2012). The most prevalent complaint of PD patients in nighttime sleep is many nightly awakes or fragmented sleep (Factor et al., 1990). PD fragmentation of sleep A lot of etiology including nightly recurrence of PD symptoms, medicine, coexisting apnea of sleep, and regular limb sleep movements (Comella et al., 2005). Patients with PD may experience nocturnal waking and difficulty sleeping (Factor et al., 1990; Happe et al., 2001). Levodopa medicament can reduce the fragmentation of sleep owing to recurring symptoms and enhance early-morning function (Askenasy and Yahr, 1985; Juncos et al., 1987; Jansen and Meerwaldtt, 1990; Pahwa et al., 1993).

#### 5.5.2 Sleep apnea

Obstructive sleep apnea was once thought to be a minor issue in PD since it was generally associated with a high BMI. Sleep apnea, on the other hand, is more frequent in PD than previously thought. Despite having a normal BMI, 20 to 50 percent of PD patients tested by sleep study had significant apnea, according to descriptive investigations (Ferini-Strambi et al., 1992; Maria et al., 2003; Braga-Neto et al., 2004). People with multiple system atrophy (MSA) have a high prevalence of respiratory problems during sleeping. MSA patients may have difficulty breathing during sleep due to vocal cord abductor paralysis (Isozaki et al., 1996; Braga-Neto et al., 2004).

#### 5.5.3 REM sleep behavior disorder

REM behavioral disorder is a sleep condition characterized by muscular movement in REM sleep with dream activity (Schenck et al., 1986; Schenck et al., 1987; Schenck et al., 2002). REm atony-free sleep (RWA) displays aberrant muscle activation without obvious behavioral behavior during REM sleep (Gagnon et al., 2002). Though RBD’s clinical characteristics seem distinct, sleep apnea can lead to a comparable condition (Iranzo et al., 2005). In patients with Basic RBD, a SPECT scan reveals a reduction in dopaminergic transport which is midway among normal controls and Parkinson patients (without parkinsonism) (Eisensehr et al., 2003).

#### 5.5.4 Excessive daytime sleepiness in PD

The typical complaint of PD-patient patients is excessive daily drowsiness (EDS). This problem has been characterized as sleep daily for PD patients on ropinirole or pramipexole, as well as a result of motor vehicle accidents recorded in 1999 (Hobson et al., 2002). Dementia and the fast advancement of parkinsonism were related to the onset of EDS (Gjerstad et al., 2002). Since its debut in the 1960s, levodopa has been known to produce drowsiness. Levodopa monotherapy produced drowsiness in the first round of 131 PD patients and restricted the dosage of levodopa in 14% of patients (Lesser et al., 1979). Although community studies indicated that almost any dopamine agonist, particularly levodopa, may cause EDS, it was shown that levodopa was the most closely connected to the disease (Hauser et al., 2000; Hobson et al., 2002).

### 5.6 Depression

Epidemiological studies are regarded as a common non-motor discovery in PD and prevalence calculations range from 2.7 percent to 70 percent. The degree of depression (moderate to serious) and the definition of depression undoubtedly influence these figures. Estimates dispute PD age and depresence, as well as the period of disease, severity of disease, and gender problems (Olanow et al., 20012001; Burn, 2002). Neurochemical and neuroimaging approaches have been appraised in the functions of dopamine, norepinephrine and serotonin, although no definite pathophysiological mechanism is recognized. A function was proposed for an allelic variant in the serotonin transporter (Mössner et al., 2001).

## 6 How natural product works on PD

### 6.1 Cellular development and apoptosis

The Bax (BCL2 associated X apoptosis regulator)/Bcl-2 (B-cell lymphoma 2) ratio, as well as caspase-3 activity, are important in apoptosis and cellular development. In this way, Bax may stop apoptosis from happening while Bcl-2 helps it happen and both caspase-3 and caspase-9 can stop Bcl-2 from doing its job. By forming the TP53-HIPK2-AXIN1 complex, HIPK2 (Homeodomain-interacting protein kinase-2) can control the creatine kinase and transcriptional activation of TP53 (Tumor protein 53). This slows down cell growth and speeds up cell death (Li et al., 2019). In cells exposed to MPP (+), Pedicularioside A inhibited the production of the caspase-3 gene as well as the cleavage of poly (ADP-ribose) polymerase (PARP) (Li et al., 2008). Pedicularioside A exerts a protective effect on mesencephalic neurons, increasing their longevity.

### 6.2 Anti-inflammatory pathway

Pathogenesis of PD is exacerbated by neuroinflammation and oxidative stress (Li et al., 2019). Some transcription factors, for instance MAFG, can promote nNOS by raising NRF1 expression. Nuclear factor erythroid-2-related factor 2 (Nrf2) protein levels and transcriptional activity, as well as overexpression of Nrf2-dependent genes, were shown to rise following therapy. A ligase modulatory component is seen in SH-SY5Y cells (Jing et al., 2016).

### 6.3 Dopamine transmission

The dopamine transporter (DAT) is in charge of DA re-uptake by altering the PP2A kinase pathway, α-synuclein inhibits VMAT2 but enhances DAT function (Dorsey et al., 2007). NURR1 expression was shown to be considerably lower in Parkinson’s sufferers, according to research, NURR1 has the ability to induce differentiation, maturity, and growth of dopaminergic neurons, protection of dopaminergic neurons, and reduction of inflammation-induced necrosis (Li et al., 2019; Rahman et al., 2022b). Immunohistochemistry results showed that treatment with ME (dried mulberry fruit extract) greatly reduced the overexpression of SNCA (Synuclein Alpha) and ubiquitin, which are two of the most important parts of LBs (Gu et al., 2017).

## 7 Herb derived Anti-Parkinson compound

The herb derived Anti-Parkinson components may produce very good activity. Some of these components are displayed in Table 1.

**TABLE 1:** Summary of some natural herbs and their mechanism of action for PD.

Compound name	Chemical structure	Plant extract	Mechanism of action	Ref
Baicalein	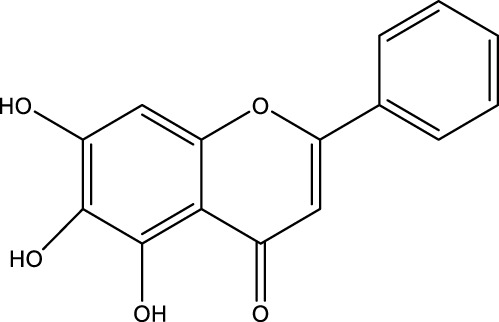	Roots of *Scutellaria baicalensis* and *Scutellarin lateriflora*	Reducing oxidative stress, inhibiting aggregation of amyloid proteins	Amro et al. (2018)
Curcumin	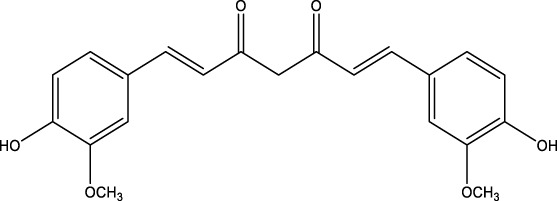	*Curcuma longa*	They have anti-inflammatory, chemotherapeutic, anti-oxidant, anti-proliferative, wound healing, and antiparasitic properties, as well as	Gupta et al. (2012)
Resveratrol	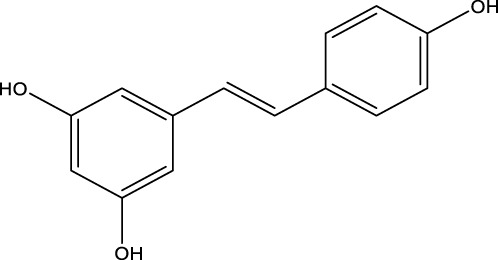	*Polygonum cuspidatum*	Improve motor impairments, oxidative stress	Lu et al. (2008)
Ginkgolides	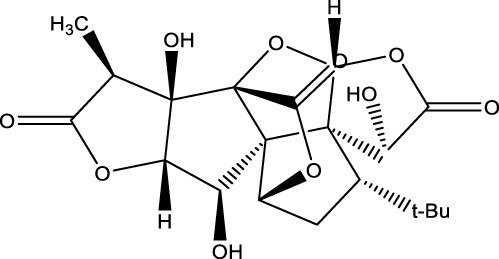	*Ginkgo biloba*	Antioxidant, reduce oxidative stress	Amro et al. (2018)
Ginsenoside	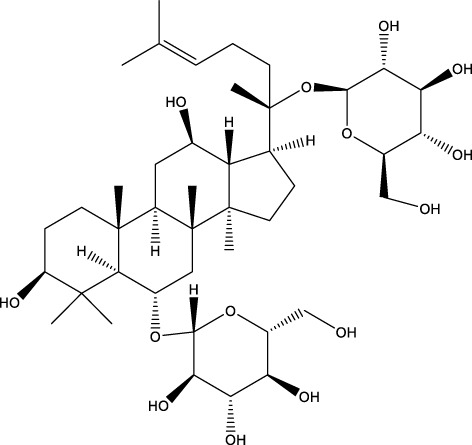	*Panax ginseng*	Antitumor, anti-inflammatory, anti-oxidation and inhibit cell apoptosis	Li et al. (2017)
L-DOPA	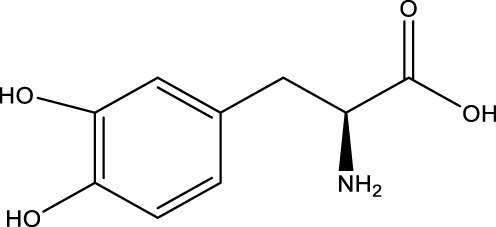	*Mucuna pruriens*	Converts to dopamine in both the CNS and periphery	Paravati et al. (2018)
Gastrodin	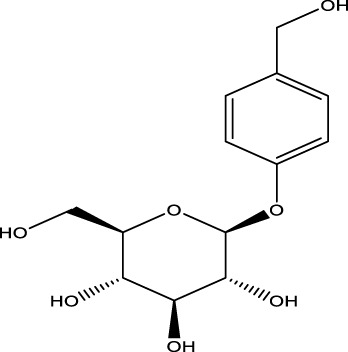	*Gastrodia elata*	Parkinson’s disease (PD), the most common type of Multiple Sclerosis (MS), is caused by the gradual death of dopaminergic neurons along the nigrostriatal pathway	Liu et al. (2018)
Nicotine	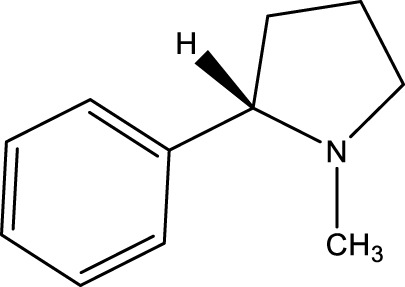	*Nicotiana tabacum*	In neural cells and brain tissue, nicotine decreases the amount of SIRT6	Nicholatos et al. (2018)
Triptolide	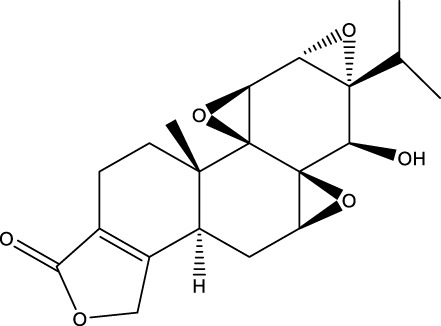	*Tripterygium wilfordii*	Anti-inflammatory and immunosuppressive effect	Yuan et al. (2019)
Paeoniflorin	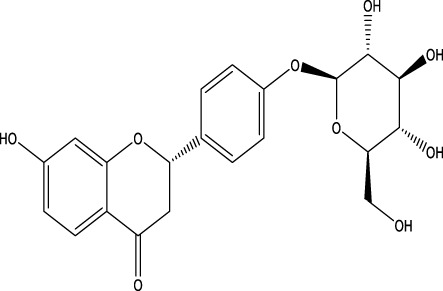	*Paeonia lactiflora*	Ca2+/calmodulin/PI3K/Akt/TNF/apoptosis signaling pathway connection, neuroactive ligand-receptor interaction, and apoptosis signaling pathway	Singh et al. (2020)
Naringenin	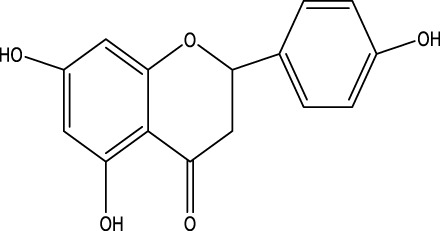	Tomatoes, grapefruits	Suppressing oxidative stress *via* antioxidant mechanism	Sugumar et al. (2019)
Terpenoids	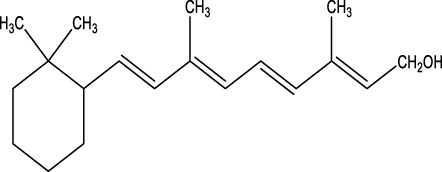	Tea, thyme, citrus food	Anti-inflammatory and neuroprotective activity	Kiyama, (2017)
Passiflora	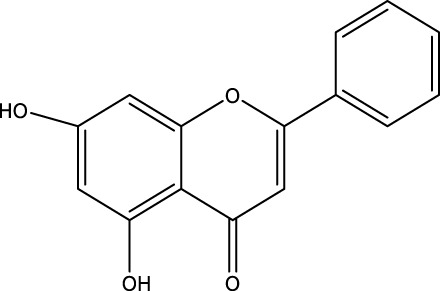	*Passion flower*	Reduced catalepsy caused by haloperidol and tacrine, indicating that it has antiparkinsonian properties	Ingale and Kasture, (2017)
Peganumharmala	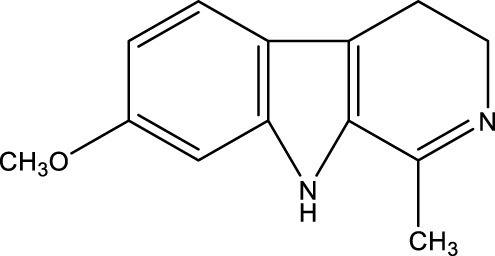	Seeds of rue	Inhibit the oxidative stress	Ingale and Kasture, (2017)
Safflower	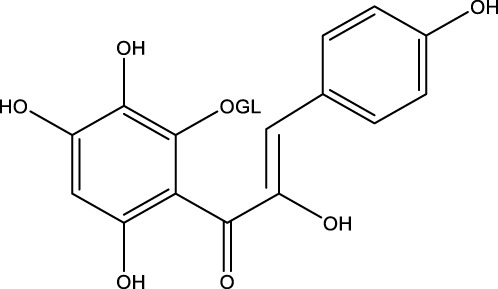	*Carthamus tinctorius*	lowered the plasma concentration of inflammatory substances and inhibited the activation of nod-like receptor protein 3 (NLRP3)	Lei et al. (2020)
St. John’s wort	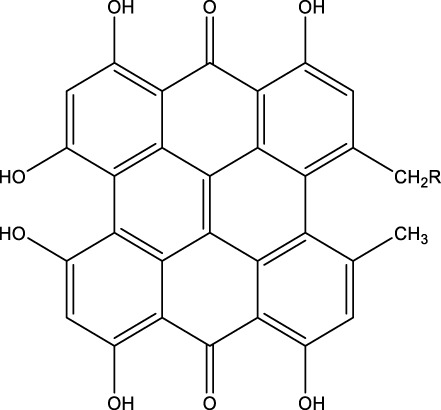	*Hypericum perforatum*	Have antioxidant effect and give antidepressant effect	Altun et al. (2013)
Fraxetin	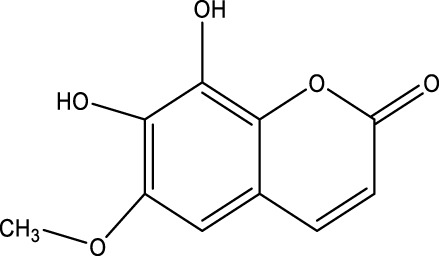	*Fraxinus bungeana*	Inhibit rotenone-induced apoptosis	Molina-Jiménez et al. (2004)

## 8 Clinical trials

### 8.1 *Mucuna pruriens* (L.) DC

Numerous laboratories are investigating the remarkable properties of the tropical legume *M. pruriens* for the treatment of PD. For the first time, a multicentric preclinical trial described the efficacy of the herbal preparation (HP-200) derived from the tropical legume for the treatment of the disease, which was administered to 60 PD patients for 12 weeks (Sengupta et al., 2016). The Hoehn and Yahr (H&Y) scale scores decreased statistically significantly, while the United Parkinson’s Disease Rating Scale (UPDRS) scores improved from the baseline level.

### 8.2 *Hyoscyamus niger* L

*H. niger* has been utilized for a variety of ailments in the traditional medical systems of many cultures, including Indian, Chinese, Roman, and Byzantine. No reference is made in the literature to its independent usage for the treatment of parkinsonism in either human patients or any animal model. However, a clinical research has confirmed its efficacy when combined with three additional Ayurvedic herbs. *H. niger* contains little L-DOPA (Nagashayana et al., 2000), but it is abundant in tropane alkaloids including hyoscine and hyoscyamine that are well known for their anticholinergic properties (Brown and Taylor, 2006). A relative excess of the neurotransmitter acetylcholine exists in the striatum due to the depletion of DA in PD, which is thought to be one of the causes of the many motor impairments linked to PD. Anticholinergics are therefore used to treat PD, especially tremor (Mirahmadi et al., 2016).

### 8.3 *Nardostachys jatamansi* DC

In a 6-OHDA-rat model of PD, an ethanolic extract of the Ayurvedic plant *N. jatamansi* roots can reduce neuronal damage (Ahmad et al., 2006). The extract effectively decreased the neurotoxin-induced lipid peroxidation, increased GSH content, the activities of GT, GR, GP, SOD, and catalase, attenuated the loss of catecholamines, increased DA-ergic D 2 receptor binding, and increased TH-immunoreactivity in the animals before 6-OHDA lesioning. *N. jatamansi* extract also dose-dependently reversed the dopaminomimetics-induced rotations and deficits in locomotor activity and muscular coordination brought on by nigrostriatal degeneration.

### 8.4 *Bacopa monnieri* L

Ayurveda makes extensive use of this plant as a brain booster. Recent preclinical studies have demonstrated its effectiveness in treating PD. *B. monnieri* extract was found to have significant preventive action in the paraquat-induced PD model in *Drosophila* and mice, principally through antioxidant capabilities and restoration of the mitochondrial ETC complexes activities (Ravikumar and Muralidhara, 2010; Hosamani et al., 2016). The ability of the plant’s alcoholic extraction to shield 6-OHDA-lesioned rats from behavioral and biochemical abnormalities were demonstrated to be significantly influenced by similar antioxidant effect (Shobana et al., 2012).

## 9 Challenges with current synthesis

When several neurotoxic models of Parkinson’s disease are put together, they make a good framework for finding anti-Parkinsonian drugs. Herbal medicines can also be used to make new Parkinson’s disease treatments. But in the future, real-world studies should look into how well plant extracts and their active parts work in PD models (Mercuri and Bernardi, 2005; Diaz and Waters, 2009). Aside from the fact that the gold standard, levodopa, does not operate on the cause, there is an additional restriction in that the symptoms become exceedingly severe after a lengthy period of therapy (Odin et al., 2008). Another significant issue with L-dopa is that it promotes neurodegeneration by causing oxidative stress (Pahwa and Lyons, 2009). While levodopa has all of these side effects, other PD medications might cause sleep difficulties and cognitive issues such disorientation and psychosis, MAO-B inhibitors, COMT inhibitors, and other similar drugs anticholinergic medications (Yuan et al., 2010). Due to great success, DBS (Deep brain stimulation) in the subthalamic nucleus (STN) and globuspallidus internus (GPi) is currently the most popular therapy (Rocchi et al., 2012; Doke et al., 2019). However, DBS has the same drawbacks in that it does not stop the disease from developing and does not prevent it from deteriorating various signs and symptoms (Tsai et al., 2009). Rehabilitative therapy is a type of treatment for PD that involves daily exercises such as stretching and muscle strengthening, exercises to strengthen and improve posture (Tomlinson et al., 2013; Clarke et al., 2016). So overall it can be said that though PD have many treatments but they have also some drawbacks that’s why these treatments are not so ideal. Further research is warranted to explore several folklore or traditional medicinal herbs and their myriad of bioactive phytochemicals to develop new safe and effective anti PD drug agents (Sharma et al., 2022a; Sharma et al., 2022b; Sharma et al., 2022c).

## 10 Conclusion and future perspectives

We discovered that several organic substances and herbal extracts display varied anti-Parkinsonian properties. When numerous PD neurotoxic models are coupled, they give a good framework for discovering anti-Parkinsonian drugs, and herbal medications can be employed to develop novel PD treatments. But in the future, real-world studies should look into how well plant extracts and their active parts work in PD models. Additionally, there is still a need for more thorough explanations of the constituents and methods of action of herbal extracts. To assure strong reproducibility, to boost therapeutic benefit, and to lessen the possibility of harm, methodological improvements must be made to upcoming clinical studies using natural products for the treatment of PD. The study design should include double blinded trials and the use of placebos. Before beginning clinical trials, protocols must be established to ensure the openness of the study findings.

### 10.1 PD vaccine

DC vaccination is a cell-based treatment. It uses antigen-loaded or sensitized DCs as the vehicle for vaccination to cause an immune response, and it is very important for getting early immune responses. T-cells become activated when they are exposed to antigen (Steinman, 1991; Boer et al., 2015). Also, peptide-sensitized DC (PSDC) vaccines cause an antigen-specific immune response that lasts longer than traditional vaccinations (Steinman, 2001). In some way, these DCs have become sensitized. They were subsequently used as a vaccination to activate immunological responses in Tg mice, which produce the 140-amino-acid full-length protein α-synuclein (B6; C3-Tg (Prnp-SNCA*A53T) human A53T variant 83Vle/J), which is controlled by the mouse prion protein promoter (Ugen et al., 2015).

### 10.2 Cell transplantation

Researchers have looked at many different cell sources to make dopaminergic neurons that can be transplanted into Parkinson’s disease patients (Lindvall, 2016). Nevertheless, current experimental and clinical evidence strongly imply that, in order to deliver significant therapeutic effect, the clinical candidate cell must be of human origin and possess the following characteristics: A neuron in the substantia nigra (Lindvall et al., 2012; Rahman et al., 2021c; Bhattacharya et al., 2022). In the first experiment to show that human ES cell-derived dopaminergic neurons can survive intrastriatal implantation and give birth to dopaminergic neurons in a rat Parkinson’s disease model, the behavior of almost all of the people improved (Roy et al., 2006). After intrastriatal transplanting, a large percentage of substantia nigra dopaminergic neurons survived for a long time, there were no tumors seen in the rodents. Furthermore, a large area of the brain was re-innervated thanks to the transplants in a bigger (nonhuman primate) brain, the striatum and behavioral impairments that resemble symptoms have improved in PD patients (Lindvall et al., 2012). Grealish and others found that grafts of human ES cell-derived dopaminergic neurons placed in a mouse were effective, axonal regeneration is possible in the rat model of PD as well as functional growth and long-term survival efficacy more than the human foetal mesencephalic dopaminergic neurons (Grealish et al., 2014). Even if no tumors have been found, safety is still an important consideration when considering human transplantation, Dopaminergic neurons generated from ES cells in patients with PD (Kriks et al., 2011; Kirkeby et al., 2012; Grealish et al., 2014; Rahman et al., 2022c).

### 10.3 Gene therapy

In 1972, gene therapy was first proposed as a way to replacing bad DNA with good DNA (Friedmann and Roblin, 1972). There are a variety of techniques, but the most common is the use of designed non-replicating viral vectors, primarily recombinant viruses of various serotypes lentivirus or adeno-associated virus (AAV) (Lang et al., 2006). Treatments with non-disease modifying medications aim to relieve the symptoms of PD by attempting to restore aberrant firing of dopaminergic or GABA-producing enzymes in the basal ganglia (Axelsen and Woldbye, 2018). So, we can expect that the gene therapy will be one the most promising therapeutic technique.

### 10.4 Surgical method

Deep brain stimulation (DBS) or localized treatments may help patients with PD enhance their quality of life and functional independence (Weaver et al., 2009). Surgical lesions and deep brain stimulation (DBS) improve medication and reduce dyskinesia as compared to standard medical therapy (Vitek et al., 2003; Weaver et al., 2009). New surgical and stimulation approaches are reshaping the sector, and technology improvements may enhance possible outcomes. Surgical decision-making is difficult, as it involves determining the best surgical candidates, among other things, such as methodology, complication management, hardware, and code changes, all of which necessitate a multidisciplinary team effort (Mitchell and Ostrem, 2020).
